# Photodynamic diagnosis of pleural malignant lesions with a combination of 5-aminolevulinic acid and intrinsic fluorescence observation systems

**DOI:** 10.1186/s12885-015-1194-0

**Published:** 2015-03-25

**Authors:** Masahiro Kitada, Yoshinobu Ohsaki, Yoshinari Matsuda, Satoshi Hayashi, Kei Ishibashi

**Affiliations:** Department of Respiratory Center, Asahikawa Medical University, Midorigaoka-Higashi 2-1-1-1, Asahikawa, Hokkaido 078-8510 Japan

**Keywords:** 5-aminolevulinic acid (5ALA), Photodynamic diagnosis, Autofluorescence imaging system

## Abstract

**Background:**

We have developed a new diagnostic method using the photosensitizer 5-aminolevulinic acid (5ALA) for diagnosing intrathoracic malignant lesions. When ingested exogenously, 5ALA is metabolized to a heme precursor, protoporphyrin IX, which stays in malignant cells and emits red to pink luminescence of about 630 nm.

**Methods:**

We enrolled 40 patients who underwent respiratory surgery and consented to participate in this study. Twenty-eight patients had primary lung cancer, 8 metastatic lung tumors, 2 malignant pleural tumors, and 2 benign tumors. Localization of malignant lesions was attempted by observing such lesions with an autofluorescence imaging system and by comparing the color tone of the autofluorescence between malignant lesions and normal tissues after oral administration of 5ALA. Malignant lesions on the pleural surface emitted pink autofluorescence in contrast to the green autofluorescence of the surrounding normal tissues.

**Results:**

When 28 patients with primary lung cancer were examined according to the degree of pleural infiltration (pl), red fluorescence was confirmed in 10 of 10 patients (100%) with p11-p13 and 5 of 18 patients (27.7%) with p10. The latter 5 patients had been diagnosed with PL1 preoperatively or intraoperatively.

**Conclusion:**

This system achieved accurate localization of malignant lesions, suggesting that it may also be applicable to photodynamic therapy.

## Background

Diagnostic imaging techniques such as computed tomography, magnetic resonance imaging, and positron emission tomography (PET), as well as visual diagnosis during surgery, are of limited value for diagnosing early malignant pleural mesothelioma or minute intrathoracic dissemination that may contribute to intrathoracic recurrence after surgery for lung cancer. Thus, a highly accurate method of evaluation and diagnosis is awaited. Focusing attention on autofluorescence, we endeavored to develop a new method of photodynamic diagnosis (PDD) using an autofluorescence imaging system. However, the initial system had drawbacks such as limitations in the visualization of lesions and unclear borders between normal tissues and malignant lesions [1]. We thus made efforts to improve the accuracy of this system. Five-aminolevulinic acid (5ALA), a photosensitizer, thereby came to our attention. Exogenous 5ALA is ingested and then metabolized to the heme precursor protoporphyrin IX, which stays in malignant cells and shows photogenesis, emitting red to pink fluorescence of about 630 nm [2]. At present, this issue is studied in the fields of neurosurgery involving brain tumors [3] and urology involving bladder and prostate cancers [4,5]_,_ but there are no reports describing the use of this technique for intrathoracic malignant lesions. In this study, we gave 5ALA orally to lung cancer patients prior to surgery, and then viewed malignant lesions using the autofluorescence imaging system. By comparing the color tone of autofluorescence between normal tissues and malignant lesions, we were able to devise a highly accurate method of localizing malignant lesions.

## Methods

### Autofluorescence imaging system

**Figure 1:**
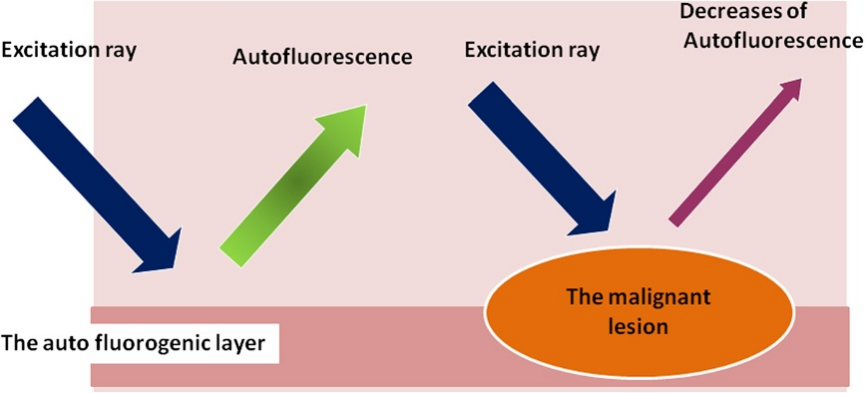
**The principle of autofluorescence observation.** Normal tissue: In response to blue excitation rays of approximately 400–450 nm, green autofluorescence of approximately 520 nm is observed. Malignant Lesion: Autofluorescence is reduced due to thickening of the mucosal epithelium, decrease in autofluorescent substances, an increase in fluorescence absorbing substances, etc., causing the color spectrum of emitted fluorescence to shift.

**Figure 2:**
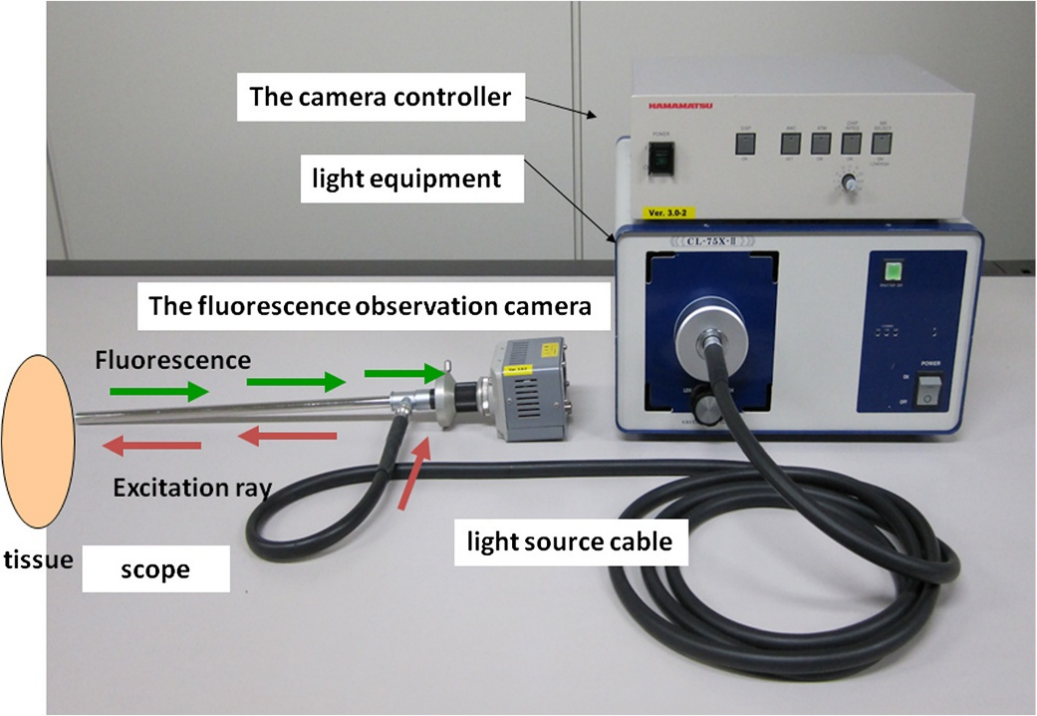
**The autofluorescence observation system.** A small CCD camera is attached to the endoscopic color fluorescence system PDS-2000, to enable white light and autofluorescence to be observed via a filter. The color fluorescence camera is equipped with a thoracoscope using the Olympus endoscopic system attachment.

Autofluorescence is spontaneous emission of fluorescence that occurs when biological structures such as mitochondria and lysosomes absorb light. The sources of autofluorescence in human tissues are collagen and fibronectin, in addition to nicotinamide-adenine dinucleotide phosphate and flavin-adenine dinucleotide [6,7]. In normal tissues, green autofluorescence of about 520 nm can be observed in response to blue excitation rays of 400–450 nm. In contrast, in cancer lesions, green autofluorescence is reduced, or the color tone of the emitted fluorescence is changed, due to thickening of the mucosal epithelium, a decrease in autofluorescent substances, or an increase in fluorescence-absorbing substances (Figure 1). The autofluorescence observation system allows autofluorescence to be observed by visualizing decreases or changes in the wavelengths of the fluorescence, and this system has already been applied clinically in the field of bronchoscopy. In this study, we endeavored to establish a method for the diagnosis of intrathoracic malignant lesions using a thoracoscope (rigid scope) equipped with the autofluorescence imaging system. The autofluorescence imaging system used in this study was shown to be an improved color fluorescence system, the PDS-2000 (Hamamatsu Photonics, Shizuoka, Japan) equipped with a small charge-coupled device (CCD) camera, allowing the observation of white light and autofluorescence via a filter [8,9]. A thoracoscope was attached to the color fluorescence camera, using an Olympus endoscopic system attachment. In addition, the LED light source, which can emit an excitation wavelength of light with a peak at 420 nm, was used (Figure 2).

### 5ALA

**Figure 3:**
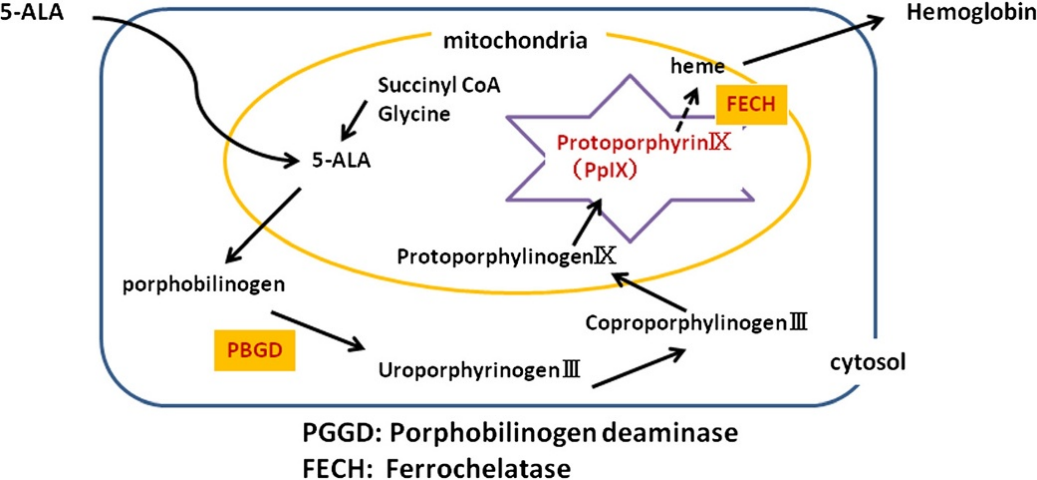
**The metabolic pathway of 5ALA.** Exogenous 5ALA is ingested and promptly metabolized to heme in normal cells. In contrast, the fluorescent substance protoporphyrin IX accumulates selectively in cancer cells, emitting red to pink fluorescence of about 630 nm, because cancer cells have high porphobilinogen deaminase activity and low ferrochelatase activity.

5ALA is the starting material in the 5-porphiline synthetic pathway, and is a natural amino acid in the human body. It is an endogenous amino acid synthesized from glycine and succinyl CoA in mitochondria, and is also a precursor of hemoglobin. When exogenous 5ALA is ingested, it is promptly metabolized to heme in normal cells. In contrast, in cancer cells, where the activity of porphobilinogen deaminase is high and the activity of ferrochelatase is low, the fluorescent substance protoporphyrin IX accumulates selectively. Consequently, red to pink fluorescence of about 630 nm is emitted (Figure 3). ALA does not stagnate at the infection part of the lung.

The subjects were patients who underwent respiratory surgery during the period between July 2013 and April 2014 and consented to participate in this study. Our system was used for a total of 40 cases: 28 cases with primary lung cancer, 8 with metastatic lung tumors, Renal cell carcinoma:1 Prostata carcinoma:1 Breast cancer:1, Colorectal carcinoma:3, Uterus cancer:1, Adenoid cystic ca of the trachea:1), 2 with malignant pleural tumors, and 2 with benign tumors (leiomyoma, nerve sheath tumors). We performed lobectomy or segmentectomy for lung cancer, partial lung resection for metastatic lung cancer, biopsies for malignant pleural tumors, and tumor resection for benign tumors. According to the degree of pleural infiltration of pathological findings (pl), the lung cancers were p10 in 18 cases, p11 in 3, p12 in 3, and p13 in 4. Exogenous 5ALA at 20 mg/kg was given orally to patients 3–4 hours before the beginning of surgery. Intrathoracic conditions were observed immediately after the initiation of surgery, employing a thoracoscope equipped with the autofluorescence observation system inserted through a 12-mm port. PL category was determined by diagnostic imaging before surgery, and pl category was determined by pathological examination after surgery. PL category is shown to the Table 1. This study was approved by the Ethics Committee of the Asahikawa Medical College. Informed consent was obtained from each patient prior to surgery.

**Table 1:** **PL category: pleural invasion of lung cancer**

PL category	
PL0	Tumor within the subpleural parenchyma, or, invading superficially into the pleural connective tissue below the elastic layer.
PL1	Tumor invades beyond the elastic layer.
PL2	Tumor invades to visceral pleural surface.
PL3	Tumor invades the parietal pleura.

Table 2 displays a summary of patient baseline characteristics. In the lung cancer patients, it showed a disease staging and pathological finding.

**Table 2:** **Patients baseline characteristics and pathology of primary lung cancer (n=28)**

	baseline characteristics and pathology
Mean age	69.1(46–79)
Gender	Men: Women: 15:13
Pathology	Adenocarcinoma :21 , Squamous cell carcinoma 5
	Large cell carcinoma: 1; Pleomorphic carcinoma: 1
pT factor	T1a/T1b/T2a/T2b/T3/T4: 12/5/4/4/1/2
PL factor	PL0/PL1/PL2/PL3: 13/8/3/4
pl factor	pl0/pl1/pl2/pl3: 18/3/3/4
p-stage	I/II/III/IV:19/6/2/1

## Results

There were no adverse events attributable to oral administration of 5ALA.

### Visualization

**Figure 4:**
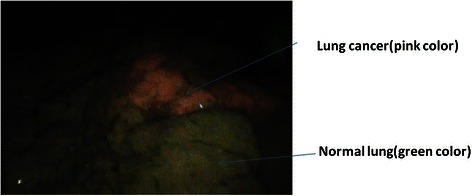
**Lung cancer (a pl1 case).** The tumor emits red light, whereas normal tissue shows green autofluorescence, providing clear borders demarcating the tumor from surrounding tissues.

**Figure 5:**
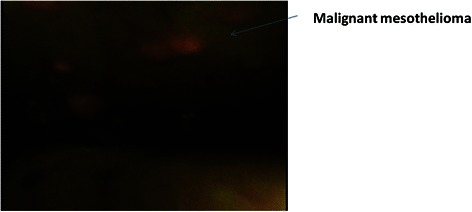
**Malignant Pleural Mesothelioma.** Red light is seen on the parietal pleural surface, consistent with the tumor.

**Figure 6:**
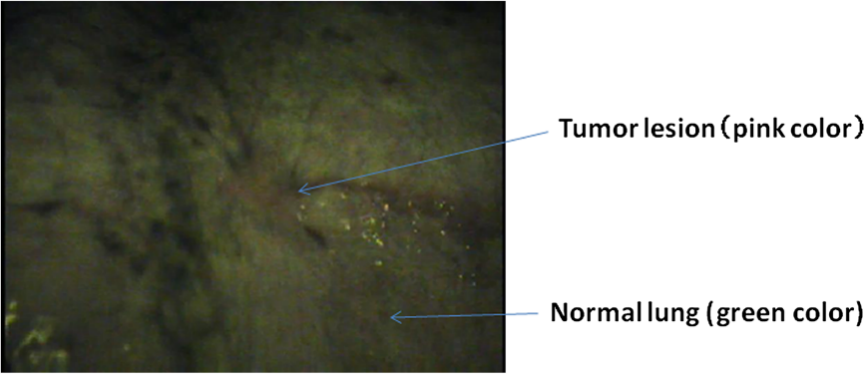
**Metastatic pulmonary tumor from renal cell carcinoma.** The tumor, although very small, emits red light, whereas normal tissue shows green autofluorescence.

**Figure 7:**
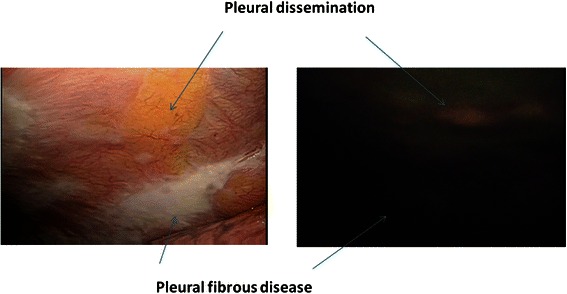
**Lung Cancer (a pleural dissemination case).** Disseminated lesions were detected during surgery under the preoperative diagnosis of a clinical T2N0 lesion. Red light is present in the area of pleural dissemination, whereas no color change is seen in the area of fibrous pleural thickening.

Lesions not distinguishable with white light could be visualized employing the autofluorescence camera; the well-defined tumor site was visualized as a pink mass in contrast to the green autofluorescence emitted by the surrounding normal tissues. Clearly demarcated lung cancer with p12 pleural invasion is shown in Figure 4. Pleural malignant mesothelioma with red to pink autofluorescence is shown in Figure 5. Metastatic lung tumors were also visualized similarly in all patients (Figure 6). Another disseminated lesion detected employing this system is shown in Figure 7. In this patient undergoing surgery based on the preoperative diagnosis of T2N0, the area of pleural dissemination was seen as red fluorescence, whereas there was no change in the color of the thickened part of the fibrous pleura.

### Visualization of red fluorescence

1. All metastatic lung tumors present on the pleural surface were visualized even when they were very small. Two pleural malignant mesotheliomas were also successfully visualized. Benign tumors (leiomyoma of the trachea and neurinoma of the mediastinum) were also observed at the time of surgery, and there were no changes in color tone in any of these cases.
2. Red fluorescence was confirmed in 15 of 28 patients (53.5%) with primary lung cancer. In terms of the degree of pleural invasion, the tumor was visualized in 10/10 (100%) cases with p11-p13. Although visualization was confirmed in 5/18 (27.7%) p10 cases, all 5 cases had been diagnosed as suspected PL1 (the degree of pleural infiltration of gross appearance or image diagnosis preoperatively). Thus, we were able to localize the lesions when there was suspected or definitive contact or infiltration of the tumor lesions into the pleura.

## Discussion

A possible causative factor in the early recurrence of lung cancer is the presence of minute disseminated lesions. Accurate diagnosis of such lesions is a very important issue in formulating optimal therapeutic strategies. Considerable attention has been given to early diagnosis of malignant pleural mesothelioma, which has a poor prognosis and has as yet no established standard treatment. It is true that there are limitations in visual diagnosis with preoperative diagnostic imaging or thoracoscopic visualization, and a diagnostic method with high accuracy is therefore desired. We have been carrying out studies focused on autofluorescence of normal tissues emitted in response to an excitation wavelength of light. We have also been striving to improve our diagnostic system, by correcting its drawbacks such as unfavorable visualization of lesions and unclear borders between normal tissues and malignant lesions [1].

Although studies on PDD using 5ALA have been reported for brain tumors, and so on, in the field of neurosurgery [3], and for bladder and prostate cancers in the field of urology [4,5], there are no reports describing such a study for thoracic malignant lesions. We have assessed PDD based on the observation of green intrinsic fluorescence emitted by normal tissue and changes in tone due to decreased fluorescent substances in malignant tumor tissue, employing our own observation system. The results showed that this system allowed us to depict actual lesions. However, because the lesion borders were blurred in some cases, the accuracy of our system required improvement. We consider the current study to demonstrate that PDD combined with 5ALA administration allows more accurate diagnosis of malignant lesions exposed on the pleural surface and that our system is effective for the detection and localization of small disseminated lesions and small metastatic tumors in lung cancer.

This technique yielded favorable results for lesions exposed on the pleura but had limitations in terms of localizing lesions without pleural invasion (pl0). In cases pathologically diagnosed as pl1 to pl3, localization was achieved with certainty. In pl0 cases, although some lesions macroscopically classified as PL1 or above could be depicted, depiction of PL0 lesions was difficult. The pleural invasion (pl) factor is emphasized in lung cancer tissue classification, and the differentiation between pl0 and pl1 lesions has important implications for lung cancer treatment guidelines and for therapeutic strategies including surgical procedures [10]. Taking these points into consideration, we have found our technique to be effective. Differentiation between benign and malignant lesions is also an important issue. In this study, there were distinct differences between malignant and benign lesions including fibrous thickening and neurogenic tumors, suggesting that our technique is applicable, to some extent, to differential diagnosis, i.e., distinguishing malignant from benign lesions. However, high standardized uptake values on PET have been reported in cases with inflammatory masses such as those of IgG4-related disease [11], underscoring the importance of further investigations in this area.

Although this study addressed the diagnosis of intrathoracic malignant disease, using 5ALA, we believe that the application of this technique is necessary not only to diagnosis but also to photodynamic therapy (PDT). In combination with heat therapy, the inhibitory effect of PDT on tumor growth was reported to be markedly enhanced by accumulation of protoporphyrin IX in tumor tissue after administration of 5ALA [12] and PDT with 5ALA achieved decreases in epidermal growth factor receptor expression and the degree of infiltration of cancer cells [13]. At present, two oncotropic photosensitizers, i.e., Photofrin (porfimer sodium) [14] and Laserphyrin (talaporfin sodium) [15], are approved for use in PDT. The principle of PDT is as follows: the photosensitizer is activated by laser light to produce active oxygen in the cellular recovery phase, resulting in attacks on malignant cells. To date, laser irradiation covering a wide area has been used to treat the malignant tumor site because oncotropic photosensitizers characteristically accumulate in malignant tumors rather than in normal tissues. However, selective localization using 5ALA may allow more selective laser irradiation, improving its therapeutic effect. This technique may also be useful as a new treatment for malignant pleural mesothelioma for which no current consensus on effective therapy exists. Studies of this issue are also underway.

## Conclusion

Photodynamic diagnosis using 5ALA for malignant intrathoracic lesions was carried out. In comparison with diagnosis using the autofluorescence observation system alone, it was possible to localize lesions based on the difference in color tone. In the future, localization of malignant intrathoracic lesions using 5ALA may allow PDT of high accuracy.

### Consent

Informed consent was obtained from each patient for publication. A copy of the written consent is available for review by the Editor-in Chief of this journal.
